# Supplementary material for: Screening of Anorectal and Oropharyngeal Samples Fails to Detect Bacteriophages Infecting Neisseria gonorrhoeae
Source: Antibiotics (Basel). 2022 Feb 18;11(2):268. doi: 10.3390/antibiotics11020268 (PMC8868155; doi:10.3390/antibiotics11020268)
Supplement: Supplementary file 1 [file antibiotics-11-00268-s001.zip › antibiotics-1589157-supplementary.pdf]

antibiotics

MDPI

Supplementary Materials

Supplementary Table S1. *N. gonorrhoeae* strains used for phage enrichment and isolation.

			Isolate Name	Year of Isolation	Location of Diagnosis	Isolation Site	MIC AZM (mg/L)	MIC CRO (mg/L)
**Phage isolation**	**Phage enrichment**	**Mix 1**	1	2019	Belgium	Pharynx	0.5	0.016
			2	2019	Belgium	Pharynx	1.5	0.016
			3	2019	Belgium	Pharynx	1	0.016
			4	2018	Belgium	Pharynx	0.125	0.023
		**Mix 2**	5	2019	Belgium	Anorectum	2	0.016
			6	2019	Belgium	Anorectum	0.19	0.125
			7	2019	Belgium	Anorectum	0.25	≤0.016
		**Mix 3**	8	2017	Belgium	Urine	16	0.03
			9	2019	Belgium	Anorectum	256	0.023
			10	2018	Belgium	Urine	1	0.19
			11	2018	Belgium	Urethra	0.25	0.19
			WHO F	1991	Canada	Unknown	0.125	<0.002
			WHO K	2003	Japan	Unknown	0.25	0.064
			WHO L	1996	Asia	Unknown	0.5	0.25
			WHO U	2011	Sweden	Pharynx	4	0.002
			WHO V	2012	Sweden	Urethra	256	0.064
			WHO W	2007	Hong Kong	Urethra or endocervix	0.5	0.064
			WHO X	2009	Japan	Pharynx	0.5	2
			WHO Y	2010	France	Urethra	1	1
			WHO Z	2013	Australia	Genital	1	0.5

MIC: Minimal Inhibitory Concentration, AZM: Azithromycin, CRO: Ceftriaxone. .
